# Variants of C-C Motif Chemokine 22 (*CCL22*) Are Associated with Susceptibility to Atopic Dermatitis: Case-Control Studies

**DOI:** 10.1371/journal.pone.0026987

**Published:** 2011-11-17

**Authors:** Tomomitsu Hirota, Hidehisa Saeki, Kaori Tomita, Shota Tanaka, Kouji Ebe, Masafumi Sakashita, Takechiyo Yamada, Shigeharu Fujieda, Akihiko Miyatake, Satoru Doi, Tadao Enomoto, Nobuyuki Hizawa, Tohru Sakamoto, Hironori Masuko, Takashi Sasaki, Tamotsu Ebihara, Masayuki Amagai, Hitokazu Esaki, Satoshi Takeuchi, Masutaka Furue, Emiko Noguchi, Naoyuki Kamatani, Yusuke Nakamura, Michiaki Kubo, Mayumi Tamari

**Affiliations:** 1 Laboratory for Respiratory Diseases, Center for Genomic Medicine, The Institute of Physical and Chemical Research (RIKEN), Kanagawa, Japan; 2 Department of Dermatology, The Jikei University School of Medicine, Tokyo, Japan; 3 Division of Otorhinolaryngology Head & Neck Surgery, Department of Sensory and Locomotor Medicine, Faculty of Medical Science, University of Fukui, Matsuoka, Fukui, Japan; 4 Department of Otorhinolaryngology Head and Neck Surgery, University of Yamanashi Faculty of Medicine, Yamanashi, Japan; 5 Takao Hospital, Kyoto, Japan; 6 Miyatake Asthma Clinic, Osaka, Japan; 7 Department of Pediatric Allergy, Osaka Prefectural Medical Center for Respiratory and Allergic Diseases, Osaka, Japan; 8 Nonprofit Organization (NPO) Japan Health Promotion Supporting Network, Wakayama, Japan; 9 Division of Respiratory Medicine, Institute of Clinical Medicine, University of Tsukuba, Ibaraki, Japan; 10 Department of Dermatology, Keio University School of Medicine, Tokyo, Japan; 11 Department of Dermatology, Graduate School of Medical Sciences, Kyushu University, Fukuoka, Japan; 12 Department of Medical Genetics, Majors of Medical Sciences, Graduate School of Comprehensive Human Sciences, University of Tsukuba, Ibaraki, Japan; 13 Laboratory for International Alliance, Center for Genomic Medicine, The Institute of Physical and Chemical Research (RIKEN), Kanagawa, Japan; 14 Laboratory of Molecular Medicine, The Institute of Medical Science, The University of Tokyo, Tokyo, Japan; 15 Laboratory for Genotyping Development, Center for Genomic Medicine, The Institute of Physical and Chemical Research (RIKEN), Kanagawa, Japan; Centre de Recherche Public de la Santé (CRP-Santé), Luxembourg

## Abstract

Atopic dermatitis (AD) is a common inflammatory skin disease caused by multiple genetic and environmental factors. AD is characterized by the local infiltration of T helper type 2 (Th2) cells. Recent clinical studies have shown important roles of the Th2 chemokines, CCL22 and CCL17 in the pathogenesis of AD. To investigate whether polymorphisms of the *CCL22* gene affect the susceptibility to AD, we conducted association studies and functional studies of the related variants. We first resequenced the *CCL22* gene and found a total of 39 SNPs. We selected seven tag SNPs in the *CCL22* gene, and conducted association studies using two independent Japanese populations (1^st^ population, 916 cases and 1,032 controls; 2^nd^ population 1,034 cases and 1,004 controls). After the association results were combined by inverse variance method, we observed a significant association at rs4359426 (meta-analysis, combined *P* = 9.6×10^−6^; OR, 0.74; 95% CI, 0.65–0.85). Functional analysis revealed that the risk allele of rs4359426 contributed to higher expression levels of *CCL22* mRNA. We further examined the allelic differences in the binding of nuclear proteins by electrophoretic mobility shift assay. The signal intensity of the DNA-protein complex derived from the G allele of rs223821, which was in absolute LD with rs4359426, was higher than that from the A allele. Although further functional analyses are needed, it is likely that related variants play a role in susceptibility to AD in a gain-of-function manner. Our findings provide a new insight into the etiology and pathogenesis of AD.

## Introduction

Atopic dermatitis (AD) is a pruritic and chronically relapsing inflammatory skin disease involving disturbed skin barrier functions, cutaneous inflammatory hypersensitivity and defects in the antimicrobial immune defense with a strong genetic background [1]. Predominant infiltration of Th2 cells is a hallmark of acute atopic AD skin lesions [2]. Most patients with AD have peripheral blood eosinophilia and increased serum IgE levels, which are reflected in an increased frequency of peripheral blood skin-homing Th2 cells producing IL-4, IL-5 and IL-13 [1]. C-C motif chemokine 22 (CCL22) and CCL17 are high-affinity ligands for CC-chemokine receptor 4 (CCR4) and induce selective migration of Th2 cells [3]. CCL22 plays a crucial role in controlling the trafficking of Th2 cells into sites of allergic inflammation and is considered to be involved in the pathology of AD [4]. Keratinocytes from patients with AD highly express thymic stromal lymphopoietin (TSLP), and CCL22 is produced by TSLP-treated dendritic cells [5]. CCL22 is upregulated in lesional atopic dermatitis skin compared with healthy skin [6], and keratinocytes in the epidermal layer of AD skin express CCL17 and CCL22 [7]. Serum levels of CCL22 in AD patients are significantly higher than those found in normal controls [8], and the levels correlate positively with disease severity in AD patients [9]. Strong positive correlations between the levels of CCL17, CCL22, and total IgE in serum of patients with AD and SCORing Atopic Dermatitis (SCORAD) have also been reported [10]. Another study reported that overproduction of IgE induced CCL22 secretion from basophils, which are essential for IgE-mediated chronic allergic dermatitis [11]. These findings prompted us to conduct an association and functional study to test whether genetic variations of *CCL22* contribute to AD susceptibility.

Several association studies using genetic variants of genes *CCL17* and *CCR4* in the CCR4 pathway have been conducted to discover genetic components in the pathogenesis of atopic dermatitis [12], [13]. A promoter polymorphism of *CCL17*, -431C>T, increases the promoter activity and the 431T allele influences higher serum levels of CCL17 [12], but genetic variants in the *CCL17* gene are not associated with susceptibility to AD. A recent study also reported that C1014T polymorphism in the *CCR4* gene was not associated with AD [13]. However, those studies were performed with small sample sizes and without replication studies. Genetic study of the *CCL22* gene has not been conducted.

In this study, we focused on the *CCL22* gene, resequenced the gene regions including all exons and introns, and carried out linkage disequilibrium mapping. We performed an association study using two independent populations and functional analyses of the related variants.

## Results

### Polymorphisms of the *CCL22* gene and LD mapping

**Figure 1:**
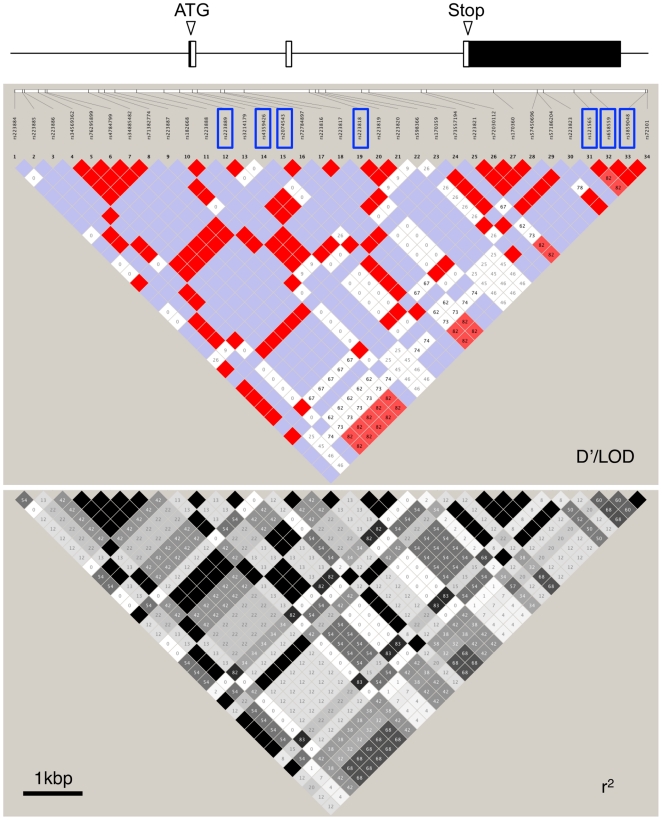
Pairwise linkage disequilibrium between 34 SNPs. LD was measured by D′/LOD (upper) and r^2^ (lower) estimated using the Haploview 4.2 program (http://www.broad.mit.edu/mpg/haploview/). Boxed variants were genotyped in this study.

We identified a total of 39 polymorphisms (Table 1). We next performed linkage disequilibrium (LD) mapping and calculated pairwise LD coefficients D′ and r^2^ among the 34 polymorphisms with MAF>10% using the Haploview 4.2 program (Figure 1). Seven tag SNPs were selected for association studies using tagger in Haploview 4.2, and these polymorphisms captured 34 of the 34 alleles with a mean r^2^ of 0.990 (r^2^>0.82). The HapMap JPT database contains genotype data for six SNPs with MAF>10% in the region (data not shown). The SNPs examined in this study covered all six SNPs shown in the HapMap JPT database.

**Table 1:** Frequencies of polymorphisms of the *CCL22* gene.

	SNP*	Location	Amino acid	MAF‡	NCBI†	
1	-3075G/A	5′-flanking region	-	0.125	rs223884	
2	-2938G/A	5′-flanking region	-	0.208	rs223885	
3	-2903T/A	5′-flanking region	-	0.333	rs223886	
4	-2668G/T	5′-flanking region	-	0.458	rs34569362	
5	-2550G/C	5′-flanking region	-	0.458	rs76295899	
6	-2511G/T	5′-flanking region	-	0.458	rs4784799	
7	-2191G/C	5′-flanking region	-	0.042	rs76720124	
8	-1795G/A	5′-flanking region	-	0.458	rs34885482	
9	-1775G/T	5′-flanking region	-	0.083	rs72784894	
10	-1618C/T	5′-flanking region	-	0.458	rs77239447	
11	-1515G/T	5′-flanking region	-	0.333	rs223887	
12	-1338A/G	5′-flanking region	-	0.208	rs182668	
13	-961G/A	5′-flanking region	-	0.208	rs223888	
14	-740A/G	5′-flanking region	-	0.083	rs3760071	
15	-488T/C	5′-flanking region	-	0.333	rs223889	§
16	-215WT/DelG	5′-flanking region	-	0.333	rs3214179	
17	5C/A	exon 1	Ala2Asp	0.125	rs4359426	§
18	88C/A	intron 1	-	0.458	rs2074543	§
19	493T/C	intron 1	-	0.458	rs72784897	
20	559G/A	intron 1	-	0.333	rs223816	
21	902C/T	intron 1	-	0.333	rs223817	
22	2030G/C	intron 2	-	0.208	rs223818	§
23	2134T/C	intron 2	-	0.208	rs223819	
24	2198T/C	intron 2	-	0.208	rs223820	
25	2314G/A	intron 2	-	0.292	rs598366	
26	2936A/G	intron 2	-	0.125	rs170359	
27	3062A/G	intron 2	-	0.458	rs73557194	
28	3766T/A	intron 2	-	0.042		
29	3970G/A	intron 2	-	0.125	rs223821	
30	4064WT/InsAAAAC	intron 2	-	0.125	rs72030112	
31	5222T/C	3′ UTR	-	0.125	rs170360	
32	5978WT/DelT	3′ UTR	-	0.125	rs57450696	
33	5979C/G	3′ UTR	-	0.375	rs57186204	
34	6089T/C	3′ UTR	-	0.125	rs223823	
35	6621A/G	3′ UTR	-	0.458	rs121565	§
36	6910G/A	3′ UTR	-	0.417	rs658559	§
37	7858C/T	3′-flanking region	-	0.458	rs3859048	§
38	7883G/A	3′-flanking region	-	0.458	rs72301	
39	8021G/A	3′-flanking region	-	0.042	rs11865093	

*Numbering according to the genomic sequence of *CCL22* (AC003665). Position 1 is the A of the initiation codon.

‡ Minor allele frequencies (MAF) in the screening population (N = 12).

† NCBI, number from the dbSNP of NCBI (http://www.ncbi.nlm.nih.gov/SNP/).

§ SNPs were genotyped in this study.

### Association of *CCL22* SNPs with susceptibility to atopic dermatitis

**Figure 2:**
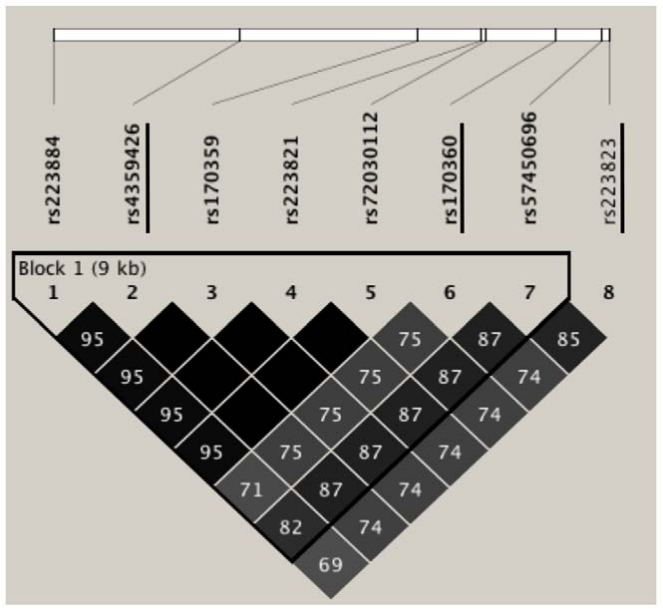
Pairwise linkage disequilibrium (r^2^) among eight SNPs in strong LD with rs4359426 in 94 control subjects. Two tag SNPs, rs170360 and rs223823, were selected for further association study. Underlined SNPs were examined.

We recruited 916 cases and 1,032 control subjects for the 1^st^ population and 1,034 cases and 1,004 control subjects for the 2nd population, respectively (Table 2). We genotyped seven tag SNPs and all genotype frequencies are shown in Table 3. The rs4359426 (A2D) SNP was associated with AD under an additive genotype model by logistic regression analysis in the first population (*P* = 0.0072; OR, 0.77; 95% CI, 0.64–0.93) (Table 3). In a replication study, rs4359426 was also associated with AD in the second population (*P* = 0.00037; OR, 0.71; 95% CI, 0.59–0.86) (Table 3). The direction of association of the SNP was similar in both of the populations. We combined the results using inverse variance method, and observed a significant association at rs4359426 (meta-analysis, *P* = 0.0000096; OR, 0.74; 95% CI, 0.65–0.85) (Table 3). We next performed further mapping analyses using two genetic variants, rs170360 and rs223823. The two SNPs were selected from among SNPs that were in strong LD (r^2^>0.87) with rs4359426 (Figure 2). Among the three variants, the strongest association was observed at rs4359426 (Table 4).

**Table 2:** Clinical characteristics of the subjects.

	Case	Control
**1^st^ population**		
Source	The University of TokyoKeio UniversityKyushu UniversityTakao Hospital	Control volunteers
Number of samples	916	1,032
Ethnicity	Japanese	Japanese
Female	43.6%	33.0%
Age (mean ± sd)	30.1±9.5	48.5±13.7
**2^nd^ population**		
Source	BioBank Japan	University of Tsukuba
Number of samples	1,034	1,004
Ethnicity	Japanese	Japanese
Female	43.8%	54.4%
Age (mean ± sd)	30.8±12.7	50.0±9.2

**Table 3:** Genotype counts and case-control association test results of seven tag SNPs.

	Allele		Case				Control			Frequency of allele 2			
db SNP ID	1/2	1/1	1/2	2/2	N	1/1	1/2	2/2	N	Case	Control	*P* value	OR (95%CI)
1st population													
rs223889	T/C	321	435	151	907	360	502	161	1023	0.406	0.403	0.82	-
rs4359426	C/A	706	191	12	909	736	269	16	1021	0.118	0.147	0.0072	0.77(0.64–0.93)
rs2074543	G/C	386	404	113	903	447	469	110	1026	0.349	0.336	0.39	-
rs223818	A/G	563	311	39	913	596	369	56	1021	0.213	0.236	0.093	-
rs121565	A/G	294	439	173	906	325	509	195	1029	0.433	0.437	0.82	-
rs658559	G/A	333	434	134	901	374	491	162	1027	0.390	0.397	0.65	-
rs3859048	C/T	399	410	103	912	466	448	108	1022	0.338	0.325	0.40	-
2nd population													
rs223889	T/C	369	497	163	1029	364	485	150	999	0.400	0.393	0.65	-
rs4359426	C/A	815	202	12	1029	722	249	22	993	0.110	0.148	0.00037	0.71(0.59–0.86)
rs2074543	G/C	404	484	133	1021	418	459	120	997	0.367	0.351	0.26	-
rs223818	A/G	647	331	42	1020	585	351	57	993	0.203	0.234	0.019	0.84(0.72–0.97)
rs121565	A/G	317	530	179	1026	317	500	180	997	0.433	0.431	0.92	-
rs658559	G/A	389	486	154	1029	363	479	148	990	0.386	0.391	0.71	-
rs3859048	C/T	425	484	117	1026	441	446	113	1000	0.350	0.336	0.35	-
Combined													
rs223889	T/C	690	932	314	1936	724	987	311	2022	0.403	0.398	0.63	-
rs4359426	C/A	1521	393	24	1938	1458	518	38	2014	0.114	0.147	0.0000096	0.74(0.65–0.85)
rs2074543	G/C	790	888	246	1924	865	928	230	2023	0.359	0.343	0.16	-
rs223818	A/G	1210	642	81	1933	1181	720	113	2014	0.208	0.235	0.0044	0.86(0.77–0.95)
rs121565	A/G	611	969	352	1932	642	1009	375	2026	0.433	0.434	0.93	-
rs658559	G/A	722	920	288	1930	737	970	310	2017	0.388	0.394	0.56	-
rs3859048	C/T	824	894	220	1938	907	894	221	2022	0.344	0.330	0.21	-

*P* values of the two populations were calculated by logistic regression analysis under an additive model. The combined *P* values were calculated using the inverse variance method. OR, odds ratio; CI, confidence interval; -, not significant.

**Table 4:** Genotype counts and case-control association test results for SNPs rs4359426, rs170360 and rs223823.

	Allele		Case				Control			Frequency of allele 2			
db SNP ID	1/2	1/1	1/2	2/2	N	1/1	1/2	2/2	N	Case	Control	*P* value	OR (95%CI)
1st population													
rs4359426	C/A	706	191	12	909	736	269	16	1021	0.118	0.147	0.0072	0.77(0.64–0.93)
rs170360	T/C	695	199	12	906	734	269	20	1023	0.123	0.151	0.011	0.78(0.65–0.95)
rs223823	T/C	728	170	11	909	765	252	10	1027	0.106	0.132	0.0093	0.77(0.63–0.94)
2nd population													
rs4359426	C/A	815	202	12	1029	722	249	22	993	0.110	0.148	0.00037	0.71(0.59–0.86)
rs170360	T/C	792	220	19	1031	728	238	26	992	0.125	0.146	0.055	0.84(0.70–1.00)
rs223823	T/C	823	189	8	1020	780	193	19	992	0.100	0.116	0.11	0.85(0.70–1.04)
Combined													
rs4359426	C/A	1521	393	24	1938	1458	518	38	2014	0.118	0.147	0.0000096	0.74(0.65–0.85)
rs170360	T/C	1487	419	31	1937	1462	507	46	2015	0.123	0.151	0.0017	0.81(0.72–0.93)
rs223823	T/C	1551	359	19	1929	1545	445	29	2019	0.106	0.132	0.0030	0.81(0.70–0.93)

*P* values of the two populations were calculated by logistic regression analysis under an additive model.

The combined *P* values were calculated using the inverse variance method. OR, odds ratio; CI, confidence interval.

### Contribution of 5′UTR rs4359426 SNP to mRNA expression levels of *CCL22*

**Figure 3:**
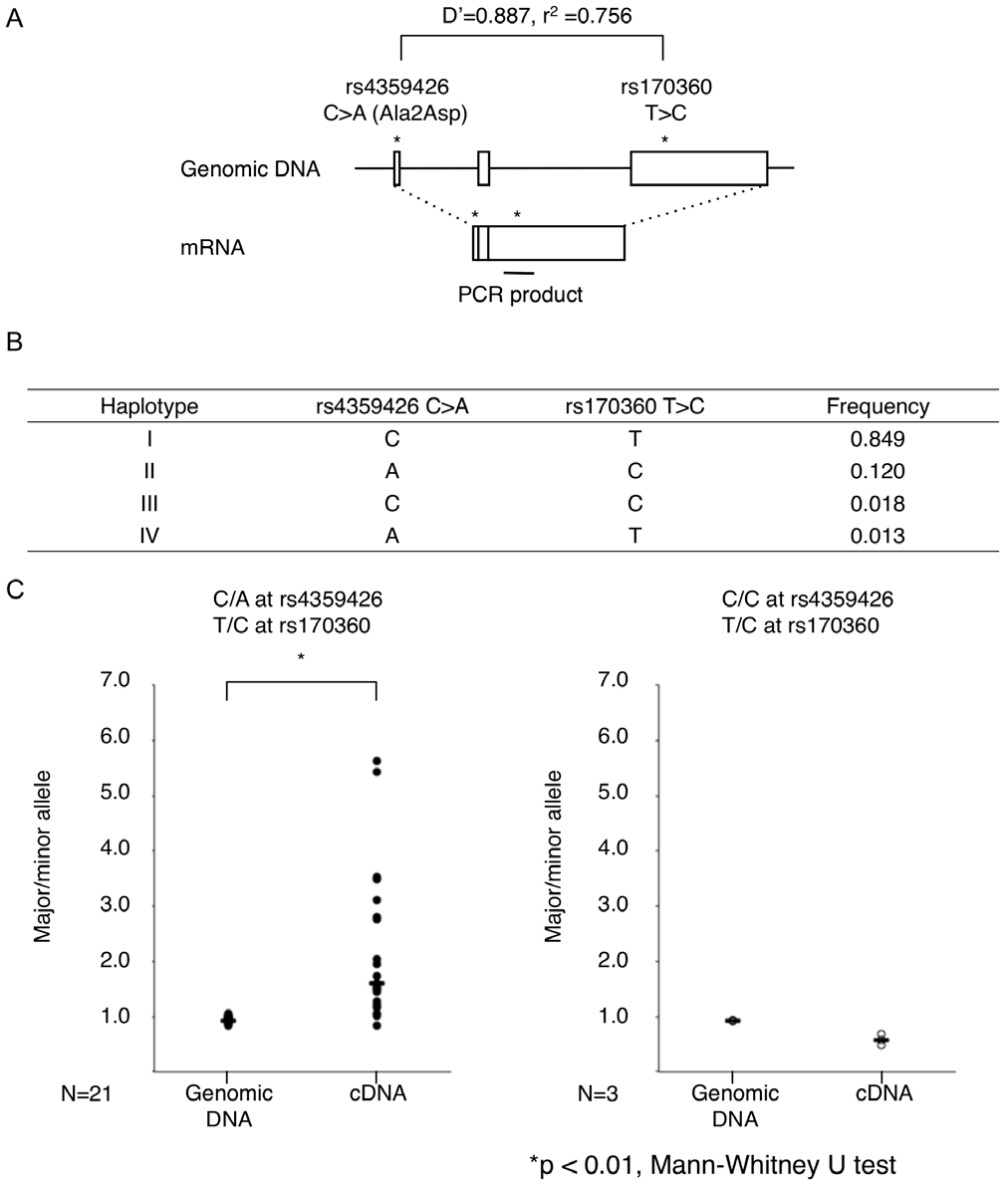
Allelic imbalance of gene expression of *CCL22* in EBV-transformed cells with heterozygous genotypes. (**A**) Genomic structures, locations and LD of the two SNPs. (**B**) Haplotypes for the two SNPs in the 1^st^ population. (**C**) The allelic ratio of PCR products from individuals. Heterozygous (left) and homozygous (right) at rs4359426. *Two-tailed *P* = 0.0000006 by the Mann-Whitney U test.

Next, using allele-specific transcript quantification (ASTQ), we evaluated whether the related variants could affect the mRNA expression level in EBV-transformed lymphoblastoid cells. As rs4359426 was located at the 16th nucleotide from the 5′ end of the *CCL22* gene (NM_002990.3), we were not able to design primers of the SNP for ASTQ analysis. We therefore designed PCR primers to encompass a SNP in the 3′-UTR of *CCL22* (rs170360) that was in strong LD with rs4359426 (Figure 3A). We isolated total RNA from 24 cell lines that were heterozygous with rs170360, and genomic DNA was used as a control for equal biallelic representation. Predicted haplotype frequencies are shown in Figure 3B. The ratio of PCR products was approximately 1.6 for cDNAs and 1.0 for genomic DNA from 21 subjects who were heterozygous for rs4359426 (Figure 3C, left panel); however, such differences were not observed in cells from three subjects who were homozygous for the C allele at rs4359426 (Figure 3C, right panel). These results implied an effect of rs4359426 and/or variants in strong LD with rs4359426 on mRNA expression levels of *CCL22*. rs4359426 and rs170360 are in absolute LD in the HapMap Caucasian populations. We further examined the expression patterns of rs4359426 and rs170360 using Genevar 3.0.2 dataset, and confirmed that the expression patterns were similar to our findings (data not shown).

### Transcription factor binding to the rs223821 SNP

**Figure 4:**
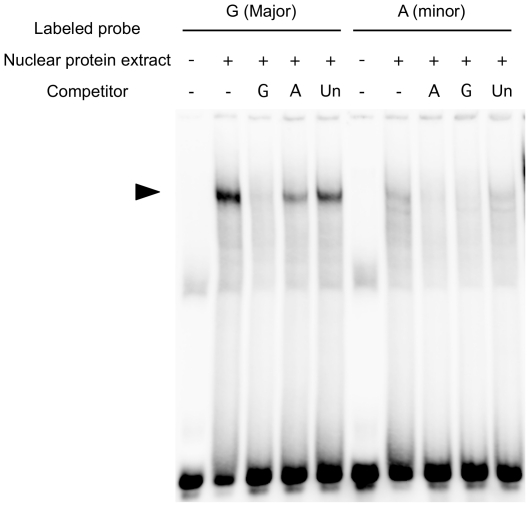
Electrophoretic mobility shift assays of rs223821. EMSA was performed using nuclear extracts from THP-1 cells stimulated with LPS (1.0 µg/ml) for 1 hour. DIG-labeled oligonucleotides corresponding to the G allele (lanes 1–5) and A allele (lanes 6–10) were used as probes. Three independent experiments were performed with similar results.

As rs4359426 was in absolute LD with rs170359, rs223821 and rs72030112 (n = 94) (Figure 2), we further examined the allelic differences of these three SNPs in the binding of nuclear proteins by electrophoretic mobility shift assay (EMSA). We could not find any specific binding of nuclear factor(s) to oligonucleotides containing rs170359 and rs72030112. However, we observed that the signal intensity of the DNA-protein complex derived from the G allele of rs223821 was higher than that from the A allele in the presence of THP-1 nuclear extract stimulated with LPS (1 µg/ml) (Figure 4). We confirmed that the complex was diminished by an excess amount of a non-labeled allele-specific competitor probe (Figure 4). This result suggested that an unidentified nuclear factor(s) interacted with the genomic region at intron 2 of *CCL22* and the SNP might have an allele-specific effect on expression through varying affinity for a transcription factor.

## Discussion

CCL22 plays an important role in the recruitment of Th2 cells into the inflammatory lesions of Th2-related diseases such as AD [14]. A recent study reported upregulation of *CCL17*, *CCL18* and *CCL22* expression in patients with AD, and suggested that the disease-specific chemokines might recruit specific memory T-cell subsets into the skin [15]. The plasma levels of CCL22 are significantly elevated in AD patients, and the values strongly correlate with disease severity [7], [10]. We identified and replicated the rs4359426 (A2D) variant of *CCL22*, which was significantly associated with AD. rs4359426 is a non-synonymous SNP and causes an amino acid substitution in the signal peptide-encoding region. We examined the influence of the amino acid substitution on the structure using SIFT (Sorting Intolerant From Tolerant) software, and the substitution at position 2 from Ala to Asp was predicted to be tolerated. In addition, no possible impacts of the amino acid substitution on the structure and function of CCL22 were predicted by PolyPhen-2 (polymorphism phenotyping v2).

Functional analyses of the related variants of *CCL22* polymorphisms showed that the susceptible allele of rs4359426 might be involved in higher mRNA expression in ASTQ analysis. We confirmed that the expression patterns from Genevar 3.0.2 dataset were similar to our findings. We also demonstrated that the genomic fragment including the risk allele of rs223821 had much higher binding affinity to the nuclear factor(s). Although it is unclear whether higher mRNA expression is influenced by altering expression enhancer activity or mRNA stability, polymorphisms in the *CCL22* gene appear to be a genetic component of the pathologic mechanisms leading to atopic dermatitis, putatively via increased *CCL22* mRNA expression.

Genetic studies reveal underlying cellular pathways, and in some cases, point to new therapeutic approaches. A recent study using a humanized model of asthma showed a critical role for DC-derived CCL17 and CCL22 in attracting Th2 cells and inducing airway inflammation [16]. In the study, administration of a CCR4-blocking antibody abolished airway eosinophilia, goblet cell hyperplasia, IgE synthesis and bronchial hyperreactivity [16]. IL-13 is an important mediator of Th2 immune responses, and there many IL-13-positive cells in AD skin lesions [17]. A recent study has shown that IL-13 induces a significant increase in the expression of CCL22 in human keratinocytes, and blocking of CCL22 in IL-13-stimulated cells results in 70–90% inhibition in migration of CD4+CCR4+ T cells [18]. These findings suggest that targeting the CCL22/CCR4 pathway might be therapeutically efficacious as a new treatment for atopic dermatitis.

The involvement of CCL22 has been reported in several immune-mediated diseases. A recent study has shown by immunohistochemistry that CCL22 is not expressed in normal skin and is markedly expressed in the lesions of atopic dermatitis, allergic contact dermatitis, and psoriasis vulgaris [19]. Another report has shown that CCL22 is present within the synovial membrane in rheumatoid arthritis and osteoarthritis patients and in high amounts in the synovial fluid of patients with rheumatoid arthritis and psoriatic arthritis [20]. To examine whether the functional SNPs found in this study are associated with those diseases will be needed for understanding of the interconnectivity of the molecular mechanisms underlying distinct diseases.

In summary, we found a significant association between susceptibility to AD and polymorphisms affecting *CCL22* expression in Japanese populations. Our findings strongly support the important role of CCL22 in AD. Although the effect of the non-synonymous SNP on protein function remains unclear, it is likely that related variants play a role in susceptibility to AD in a gain-of-function manner. Further functional analyses and replication studies in other populations are needed; however, our findings provide insights into the pathophysiology of AD.

## Materials and Methods

### Subjects

A total of 1,950 case subjects with AD were recruited from several hospitals as described [21]. Case subjects in a second population were obtained from the BioBank Japan [22]. All case subjects were diagnosed according to the criteria of Hanifin and Rajka [23]. A total of 1,032 control volunteers in the first set who had no history of AD were recruited by detailed physicians' interviews. For the second set, a total of 1,004 controls who had never been diagnosed with AD were recruited during their annual health checkup in the University of Tsukuba (Table 2). All individuals were Japanese and gave written informed consent to participate in the study. This research project was approved by the ethics committees at the Institute of Medical Science, the University of Tokyo and the RIKEN Yokohama Institute.

### Resequencing of the *CCL22* gene and genotyping

We first resequenced the *CCL22* region to identify genetic variations using DNA from 12 subjects with AD. We surveyed the gene from 3 kb of the 5′ flanking region to a 1 kb continuous 3′ flanking region of the last exon on the basis of genomic sequences from the NCBI database (NC_000016.9). The PCR product was reacted with BigDye Terminator v3.1 (Applied Biosystems), and sequences were assembled and polymorphisms identified using the SEQUENCHER program (Gene Codes Corporation, Ann Arbor, MI).

Genotyping of the seven SNPs in *CCL22* was performed by the TaqMan™ allele-specific amplification (TaqMan-ASA) method (Applied Biosystems) and multiplex-PCR based Invader assay (Third Wave Technologies).

### Allele-specific transcript quantification (ASTQ)

We conducted allelic expression analyses by TaqMan assay using SNP genotyping probes as described [24]. EBV-transformed lymphoblastoid cells were obtained from the Health Science Research Resources Bank of Japan. Genomic DNA was used as a control for equal biallelic representation. The allelic ratio for each cDNA and genomic DNA was measured.

### Electrophoresis Mobility Shift Assay

EMSA was performed using nuclear extracts from THP-1 cells stimulated with LPS (1.0 µg/ml) for 1 hour. DIG-labeled oligonucleotides corresponding to the G allele (lanes 1–5) and A allele (lanes 6–10) were used as probes. The oligonucleotide sequences were 5′-ATCGCCTGAACCCGGGAGTTGGAGGTT for the G allele and 5′-ATCGCCTGAACCCAGGAGTTGGAGGTT for the A allele. For competition, a 100-fold excess of unlabeled G or A allele oligonucleotides or unrelated oligonucleotides (Un) (TFIID) was used.

### Statistical analysis

We calculated allele frequencies and tested agreement with Hardy-Weinberg equilibrium using a chi-square goodness-of-fit test. We then compared differences in the allele frequencies between case and control subjects by logistic regression analysis under an additive model and calculated odds ratios (ORs) with 95% confidence intervals (CIs). Results for the 1st and 2nd populations were combined by fixed effect inverse-variance method using Genome-Wide Association Meta Analysis (GWAMA, http://www.well.ox.ac.uk/gwama/tutorial.shtml) [25]. We applied Bonferroni corrections, the multiplication of *P* values by the number of variants investigated. Corrected *P* values of less than 0.05 were judged to be significant. The expression patterns of SNPs were obtained from Genevar (GENe Expression VARiation) 3.0.2 (Wellcome Trust Sanger Institute). We examined the influence of amino acid substitution on the structure using SIFT software (http://sift.jcvi.org/) and PolyPhen-2 (polymorphism phenotyping v2) (http://genetics.bwh.harvard.edu/pph2/).
